# Polysubstance-induced relapse of schizoaffective disorder refractory to high-dose antipsychotic medications: a case report

**DOI:** 10.1186/s13256-016-1031-3

**Published:** 2016-09-06

**Authors:** Murray G. Tucker, Sebastian Kekulawala, Michelle Kent, Sam Mostafa, Richard Harvey

**Affiliations:** 1 Mental Health, Drugs and Alcohol Service, Barwon Health, University Hospital Geelong, Geelong, Victoria Australia; 2 GenesFX Health, North Melbourne, Victoria Australia; 3 School of Medicine, Deakin University, Waurn Ponds, Victoria Australia

**Keywords:** Methamphetamine, Substance-induced psychosis, Psychiatric emergencies, Rapid sedation, Medication safety, Pharmacogenetic testing

## Abstract

**Background:**

The high prevalence of comorbid illicit drug use in persons with chronic psychotic illness represents a strong determinant of psychotic relapse and rehospitalization. Epidemiological studies indicate changing patterns of illicit drug use in Australia, which are concerning because of increased use of crystal methamphetamine, also known as “ice.” An important complication of habitual use of crystal methamphetamine is the development of a dose-dependent acute psychotic reaction. We report a case of an acute psychotic relapse in response to polydrug use most notable for multiple recent binges of crystal methamphetamine. Unlike previously described case reports, our patient’s acute psychosis was refractory to ultra-high doses of multiple antipsychotic medications. This presented safety challenges due to the risk of serious side effects with high-dose antipsychotic medications.

**Case presentation:**

A 30-year-old white man with a past history of schizoaffective disorder was brought to our emergency department by the police in a state of extreme agitation, combativeness, and paranoia after use of cannabis and crystal methamphetamine. Despite existing compliance with zuclopenthixol decanoate depot medication, he required multiple emergency injections of zuclopenthixol acetate, and regular high-dose droperidol, chlorpromazine, and lorazepam. However, he remained severely agitated and psychotic with continuous threats of harm to others. A test of antipsychotic drug metabolism by cytochrome P450 enzymes did not reveal a pharmacogenetic cause for the poor therapeutic efficacy of antipsychotic medications. His psychosis did not appear to be modified by psychoactive medications but was instead self-limited to the presence of endogenous methamphetamine within his system. He fully recovered 96 to 120 hours post-presentation and was discharged home with out-patient clinic follow-up.

**Conclusions:**

The current case highlights the challenging nature of a severe psychotic relapse precipitated by illicit substances that is resistant to medical management. High doses of multiple antipsychotic medications may be required to manage dangerous behaviors associated with these acute psychotic relapses. These patients require close monitoring for adverse effects with adjustment of dosing to ensure the optimal balance of risk versus benefit while the patient is acutely psychotic. The results are of relevance for the management of psychiatric emergencies in emergency departments and acute mental health settings.

## Background

Illicit drug use is a significant problem among persons with major mental illness. The 2010 Australian National Survey of Psychotic Disorders reported psychoactive substance abuse in 63 % of men and 41 % of women with psychosis, compared with 12 % of men and 6 % of women in the general population [1]. This high comorbidity represents one of the biggest barriers to effective management of schizophrenia and related disorders since substance use can reduce compliance with medications, exacerbate psychosis, precipitate a major relapse of illness requiring hospitalization, and increase treatment resistance over the lifetime of the illness [2, 3]. It has been hypothesized that the high comorbidity of illicit drug use and chronic psychotic disorders may reflect an inherent neurobiological vulnerability to developing a substance abuse disorder, a strategy to alleviate symptoms of the primary mental illness or the adverse effects of medications, or that patterns of use may simply reflect the local availability of illicit substances [4].

The illicit drugs of choice for persons with major psychotic disorders are most frequently cannabis followed by stimulants such as cocaine, amphetamine, and methamphetamine. Approximately 11 % of persons with schizophrenia abuse cannabis and stimulants concurrently [2]. One psychostimulant of particular interest is crystalline methamphetamine, also known as “ice,” which is both highly addictive and growing in popularity. Ice use reportedly more than doubled from 22 % to 50 % among illicit amphetamine users in Australia between 2010 and 2013 [5]. The frequency of daily or weekly ice use also doubled from 12.4 % to 25 % over the same period [5]. Correspondingly there have been increased public concerns about the drug’s detrimental impact on mental and physical health, social functioning, crime rates, and public safety since intoxicated users have a propensity towards hostility and interpersonal violence [6]. Escalation in the use of ice has thus resulted in a predictable rise in methamphetamine-related presentations to Australian emergency departments [6, 7].

A relatively common illness associated with habitual or binge use of methamphetamine is a transient psychotic reaction. For individuals without a history of primary psychotic illness, this psychotic reaction is referred to as methamphetamine-induced acute psychosis. Comparatively, for individuals with a pre-existing primary psychotic disorder, this psychotic reaction is referred to as an acute psychotic relapse precipitated by methamphetamine abuse. The clinical features of methamphetamine-induced acute psychosis commonly include hallucinatory experiences and persecutory delusions accompanied by hostile behavior [8]. Bizarre delusions, formal thought disorder, or negative symptoms are less common [8–10]. Consequently, methamphetamine-induced acute psychosis can appear remarkably similar to acute paranoid schizophrenia [11]. The time course of methamphetamine-induced acute psychosis is normally brief, lasting hours to a few days, with patients usually making a full recovery with abstinence [12]. It has been reported that patients with methamphetamine-induced acute psychosis normally respond well to antipsychotic medications [13]. However, there have been very few reports of severely unwell patients with either methamphetamine-induced acute psychosis or methamphetamine-precipitated psychotic relapse who respond poorly to antipsychotic medications and the safety issues that arise in this scenario.

## Case presentation

A 30-year-old white man was brought to our emergency department by the police under the Mental Health Act in an aggressive and combative state threatening suicide and homicide. His presentation was precipitated by daily cannabis use and multiple binges of ice over the prior month. His past psychiatric history included childhood attention deficit hyperactivity disorder (ADHD) and schizoaffective disorder which was managed on a Community Treatment Order with fortnightly 300 mg zuclopenthixol decanoate intramuscular injections. His past medical history was remarkable for polysubstance abuse. From 15 years of age he regularly used tobacco, alcohol, and cannabis, and sporadically used heroin, hallucinogens, ecstasy, and amphetamines. In terms of family history, his father had ADHD and one historical episode of manic psychotic illness requiring treatment with medication and electroconvulsive therapy. His male sibling was deceased from suicide after protracted illness with major depression and binge eating disorder. Our patient had a long history of transient living and difficulty sustaining regular employment in bricklaying. At the time of presentation, he was living in a shared residence and using ice most days in the context of interpersonal conflict, unemployment, financial stressors, and housing stressors.

On his arrival at our emergency department, six-point mechanical restraint was required for his safety and for the safety of the staff and co-patients. An initial physical examination revealed Glasgow Coma Score of 14 (Eyes 4, Voice 4, Motor 6), tachycardic pulse 110 beats/minute, blood pressure 125/63 mmHg, fingertip oxygen saturation 95 % on room air, and tympanic temperature 36.1 °C. A subsequent physical examination revealed that his pupils were equal and reactive to light, heart sounds were dual with nil added sounds or murmurs, chest auscultation was normal bilaterally, abdomen was soft and non-tender, and upper and lower limbs were neurologically intact based on gross examination. An electrocardiogram could not be recorded due to his combative behavior. He claimed that he had been stabbed in the torso by his flatmate despite no evidence of any external injuries. A full blood examination, C-reactive protein test, random blood glucose test, liver function tests, thyroid function tests, and ethanol level were unremarkable. Renal function tests revealed mild hypokalemia, his potassium (K+) was 3.2, and mild acute kidney injury, his creatinine was 126 and estimated glomerular filtration rate (eGFR) was 66, secondary to poor oral intake. His dehydration was treated with 1 L of 0.9 % normal saline solution administered intravenously due to his refusal to take food and fluids orally.

Rapid sedation was commenced with ziprasidone, lorazepam, droperidol, and zuclopenthixol acetate (Table 1). Benztropine was administered for prophylaxis against extrapyramidal side effects of the antipsychotic medications. The level of sedation attained was unsatisfactory as he remained severely agitated and combative interspersed with only brief periods of drowsiness. He could not follow direction or adhere to boundaries established by staff. All attempts at de-escalation and distraction were met with aggression.

**Table 1:** Medications administered to the patient in our emergency department and selected behavioral observations

Time	Medication name, dose, and route	Behavioral observations
1720	Approximate time of presentation to our emergency department	Extremely aggressive, threatening, and offensive behavior and language
1726	Ziprasidone 20 mg IM, lorazepam 2 mg IM	
1730	Lorazepam 2 mg IM	
1740	Zuclopenthixol acetate 150 mg IM, benztropine 2 mg IM	
1805	Lorazepam 2 mg IV	Vital signs and IV access obtained, blood sampled
1830	Droperidol 10 mg IM	Verbally abusive, threatening, aggressive
2230	Ziprasidone 20 mg IM	
2300	Lorazepam 2 mg IV	
0100		Sedated and quiet
0400	Droperidol 10 mg IM, Lorazepam 2 mg IV	Yelling, abusive, shaking bed, threatening staff
0600		Sedated but intermittent abuse and threats
1120	Ziprasidone 20 mg IM	
1300	Droperidol 25 mg IM	Acute arousal, combative during transport to psychiatric ward

*IM* intramuscular, *IV* intravenous

After psychiatric review in our emergency department he was transferred to a closed seclusion room on the psychiatric ward. He scored maximally on the Dynamic Appraisal of Situational Aggression (DASA) scale [14]. Droperidol 25 mg and lorazepam 2 mg were administered intramuscularly four times a day under physical restraint. His vital signs were measured every 4 hours; they remained within normal limits and no extrapyramidal side effects were observed.

A psychiatric review was re-attempted 27 hours into closed seclusion. However, this review was impossible because he remained extremely agitated and uncooperative. Interactions with our patient during seclusion checks revealed an absence of perceptual disturbances, thought disorder, or delusions of reference. He was no longer complaining of being stabbed in the torso, but continued to express paranoid thought content. A urine drug screen was positive for methamphetamine, benzodiazepines, and tetrahydrocannabinol. A recheck of his renal function was not performed because he was now consuming regular food and fluids and his urine output was normal.

After 72 hours of emergency psychiatric treatment, high-dose antipsychotic medications administered intramuscularly remained ineffective. He was becoming slightly more cooperative with nursing staff requests despite his continued aggression. Intramuscular administration of droperidol was ceased and oral administration of chlorpromazine syrup 300 mg four times a day and lorazepam 2 mg four times a day was commenced (Table 2). A second prophylactic dose of benztropine was also administered at this time. An urgent electrocardiogram revealed that his QT-corrected interval was normal (432 ms).

**Table 2:** Medications administered every 24 hours since presentation to our emergency department

Time since presentation to emergency department	Total medication doses over 24-hour period	Daily chlorpromazine equivalence	Daily diazepam equivalence
0–24 hours	Lorazepam IV/IM 10 mg		50 mg
	Ziprasidone IM 60 mg	300 mg	
	Droperidol IM 45 mg	900 mg	
	Zuclopenthixol acetate IM 150 mg	300 mg	
	Benztropine IM 2 mg		
24–48 hours	Lorazepam IM 6 mg		30 mg
	Droperidol IM 100 mg	2000 mg	
	Zuclopenthixol acetate IM 150 mg	300 mg	
48–72 hours	Lorazepam IM 8 mg		40 mg
	Lorazepam PO 2 mg		10 mg
	Droperidol IM 50 mg	1000 mg	
	Zuclopenthixol acetate IM 100 mg	200 mg	
	Chlorpromazine PO 300 mg	300 mg	
72–96 hours	Lorazepam PO 16 mg		80 mg
	Chlorpromazine PO 1350 mg	1350 mg	
	Zuclopenthixol acetate IM 100 mg	200 mg	
	Benztropine PO 2 mg		
96–120 hours	Lorazepam PO 10 mg		50 mg
	Chlorpromazine PO 750 mg	750 mg	
	Zuclopenthixol decanoate IM 300 mg	450 mg^a^	
	Benztropine PO 2 mg		

Daily chlorpromazine and diazepam equivalence are provided for comparative purposes [31]. *IM* intramuscular, *IV* intravenous, *PO* oral. ^a^Zuclopenthixol decanoate depot of 300 mg/fortnightly is equivalent to 450 mg of chlorpromazine daily over the fortnight

His DASA score remained maximal despite his acceptance of oral medications. His behavior was marked by severe aggression, hostility, threats to kill, punching and kicking of the seclusion door, periods of loud abuse, impulsivity, and unpredictability. This behavior continued despite treatment which consisted of high-dose antipsychotic medications and lorazepam for 3 days. An urgent application was made to the Mental Health Tribunal for electroconvulsive therapy to treat his psychotic relapse. This application was subsequently withdrawn when there was a sudden improvement in his mental state at 96 hours of treatment. His aggression settled to a DASA score of 4 out of 7 with only low-level irritability and no further threats of suicide or homicide. A repeat urine drug screen revealed a trace amount of methamphetamine.

He was engaged and cooperative for the first time at review 120-hours post-presentation. He reported that he felt threatened by people who he alleged were trying to stab him. However, he was able to acknowledge that his paranoia was possibly caused by a relapse of his mental illness. He denied any psychotic or affective symptoms, or any thoughts, intent, or plans to harm himself or others. There were no signs of major mental illness that required ongoing in-patient treatment, and he was discharged home on a Community Treatment Order with continued zuclopenthixol decanoate depot medication. After discharge, he engaged regularly with our mental health service to receive his depot. He continued to use cannabis daily and crystal methamphetamine once or twice per week. His mental state remained stable during out-patient psychiatric reviews with occasional vague auditory hallucinations. He had no further admissions to acute psychiatric units during a period of 18 months post-discharge.

## Discussion

The current case highlights the difficulty of managing severe agitation in acutely psychotic patients whose symptoms are refractory to antipsychotic medications. The patient experienced a psychotic relapse of his schizoaffective disorder which was precipitated by polydrug use, most notable for daily cannabis use and multiple binges of crystal methamphetamine. A strong relationship between heavy methamphetamine use and the development of acute psychotic illness is well documented [11]. In those with chronic psychotic illness, comorbid illicit amphetamine use can exacerbate active psychosis or cause a relapse of psychotic illness that was previously in remission [8]. This observation has been verified in studies which experimentally administered a small dose of a stimulant drug to patients with schizophrenia and elicited a dramatic increase in positive psychotic symptoms and occasionally a reduction in negative symptoms [15]. Exacerbation of psychotic illness by stimulants is not necessarily prevented by existing compliance with antipsychotic medications [3, 15], which was observed in our patient since he was compliant with fortnightly 300 mg zuclopenthixol decanoate injections prior to his relapse.

**Fig. 1:**
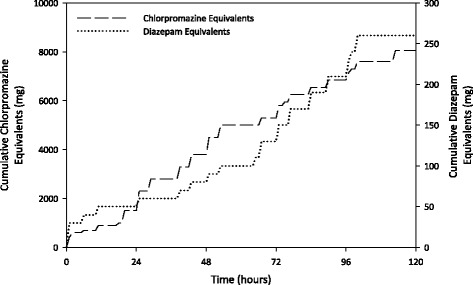
Cumulative chlorpromazine and diazepam equivalents for antipsychotic and benzodiazepine medications respectively that were administered to the patient

Currently there are no clear guidelines for the management of acute psychosis precipitated by illicit drugs [16]. Antipsychotic medications, with or without benzodiazepines, are normally effective in standard doses and are widely used [17]. A prospective randomized study [13] administered 2 to 4 mg of lorazepam or 2.5 to 5 mg of droperidol intravenously to 166 severely agitated persons presenting to an emergency department with methamphetamine-induced acute psychosis or methamphetamine-precipitated relapse of a psychotic disorder. The patients’ behavior generally settled to cooperative, somnolent, or easily roused within 30 minutes of administration of either medication. In contrast, our patient was administered standard doses of lorazepam, ziprasidone, zuclopenthixol acetate, and droperidol within the first hour, yet he remained dangerously hostile and combative (see Table 1). Other prospective randomized studies have reported that either regular olanzapine and haloperidol [18], quetiapine and haloperidol [19], or aripiprazole and risperidone [20] produce clinically significant reductions in psychotic symptomatology in cases of amphetamine-induced psychosis. Therefore, the current case is remarkable for severe psychotic agitation that was refractory to ultra-high doses of multiple antipsychotic medications and benzodiazepines (see Fig. 1 for cumulative chlorpromazine and diazepam equivalents). The medications did not seem to alter the clinical course of his relapse. Rather, his relapse appeared to be self-limited to the clearance of the endogenous methamphetamine.

To maximize patient safety, physical health checks occurred at 4-hourly intervals to monitor his vital signs, mental state, and medication adverse effects. His hostility made these physical health assessments particularly difficult. Large and repeated doses of benzodiazepines may cause hypotension and respiratory depression; however, this was not observed as his vital signs remained within normal limits. Droperidol is a high potency antipsychotic medication which can cause extrapyramidal side effects of akathisia and acute dystonic reactions (for example, torticollis, oculogyric crisis). These side effects were prevented by administering multiple prophylactic doses of benztropine. Antipsychotic medications and methamphetamine may also prolong the QT interval which increases the risk of life-threatening arrhythmias. A baseline electrocardiogram could not be recorded due to combative behavior. However, an electrocardiogram was later recorded when he became more cooperative which showed that his QT interval was normal. Given the refractory nature of his psychosis to medical management and the high risk of adverse effects of high doses of antipsychotic medications, we considered electroconvulsive therapy; clinicians may opt for electroconvulsive therapy at an earlier stage in similar psychiatric emergencies. Electroconvulsive therapy alone or in combination with antipsychotic medications is recommended for treatment-resistant psychotic disorders when a rapid clinical response is urgently required [8]. Preliminary studies also indicated that electroconvulsive therapy can induce remission of persistent symptoms of methamphetamine-induced psychosis [21, 22].

We hypothesized that our patient’s poor clinical response was from a genetic polymorphism in the drug-metabolizing activity of cytochrome P450 enzymes. Enzyme CYP2D6 metabolizes up to 25 % of commonly prescribed drugs including antipsychotic medications such as chlorpromazine, haloperidol and zuclopenthixol [23]. “Ultra-rapid metabolizers” metabolize antipsychotic medications at a higher rate than the general population and require more medication for therapeutic effects [23]. After he had recovered he provided informed consent for a buccal swab of cheek cells for pharmacogenomic testing. The results of the test were two normal functioning alleles of the CYP2D6 enzyme, which is an “extensive metabolizer” phenotype (that is, normal in the general population), thus refuting our hypothesis. Alternative mechanisms underlying refractoriness to antipsychotic treatment may include drug interactions, unknown contaminants within clandestine laboratory-synthesized methamphetamine, and induction or inhibition of CYP450 enzymes. For instance, our patient smoked cigarettes which induce the production of CYP1A1, CYP1A2, and CYP2E1 enzymes. Induction of these enzymes can lower the plasma concentrations of several psychotropics including chlorpromazine and benzodiazepines [24]. Benztropine has also been reported to exacerbate psychosis in persons with schizophrenia who were previously well managed on antipsychotic medications; however, this effect is controversial [25].

Degenerative changes in a patient’s dopaminergic pathways may also account for a suboptimal effect of antipsychotic treatment. Chronic amphetamine abuse is known to cause enduring structural and functional changes in dopaminergic systems, which reduces the efficacy of antipsychotic medications [26]. Lieberman and colleagues [27] also highlighted that 30 to 60 % of patients with schizophrenia develop increasing resistance to standard antipsychotic medication over the natural course of their illness for reasons that are poorly understood but may involve sensitization to dopamine or degeneration of dopamine neurocircuitry. Genetic polymorphisms in dopaminergic receptors and transporters have also been identified as risk factors for ADHD and schizophrenia and are thought to contribute to the dysfunctional dopamine signaling underlying these illnesses [28, 29]. Moreover, this is the major signaling system targeted by antipsychotic drugs. Of interest, our patient’s father had comorbid ADHD and manic psychotic disorder, and our patient’s male sibling had major depression and binge eating disorder. Taken together, this suggests a strong genetic contribution of dysfunctional dopaminergic signaling to account for our patient’s psychiatric illnesses, as well as the refractory nature of his psychosis to treatment.

By convention, the technically correct diagnosis in this case was an acute psychotic relapse of our patient’s schizoaffective disorder which was precipitated by polydrug use. However in many respects his presentation appeared to be classical for methamphetamine-induced acute psychosis, which is a diagnosis normally reserved for individuals without an underlying primary psychotic disorder. That is, he presented in a state of clear consciousness with non-bizarre persecutory delusions that his flatmate had stabbed him and was trying to kill him. His affect was congruent with the content of his paranoid beliefs and there was no evidence of formal thought disorder. He was severely agitated with persistent anger and threatening behavior which seemed to stem from paranoia of others wanting to harm him in combination with poor insight, impulsivity, and anti-social personality traits. His psychotic symptoms also closely followed the timeline of methamphetamine intoxication since a urine drug screen performed at the time of his recovery revealed the clearance of methamphetamine from his system. In contrast, his previous exacerbations of schizoaffective disorder were characterized by a different symptomatology marked by auditory hallucinations, bizarre persecutory delusions (for example, being haunted by spirits), “pressure of speech,” and flight ideas which lasted for several weeks. This is an interesting feature of the case and suggests that it may be possible to distinguish between drug-induced psychotic states and alternative mechanisms causing a relapse of manic psychotic illness when the patient’s past psychiatric history is accurately known [8, 30]. However, more research is required to clarify this issue.

## Conclusions

Our patient experienced a particularly severe acute psychotic relapse which lasted 96 to 120 hours and was precipitated by daily cannabis and binges of crystal methamphetamine. His acute psychosis was marked by continuous severe hostility which was unusually resistant to ultra-high doses of multiple antipsychotic and benzodiazepine medications. In these treatment-refractory cases, there is a significant potential for harm to the patient, staff, and the public. Risks to the patient include self-harm, suicide, and adverse effects of medications. Although the adverse effects of psychoactive medications are difficult to monitor in uncooperative patients they can be life threatening and thus warrant special attention.

## Abbreviations

ADHD: Attention deficit hyperactivity disorder
DASA: Dynamic Appraisal of Situational Aggression
eGFR: Estimated glomerular filtration rate
IM: Intramuscular
IV: Intravenous
PO: Per oral
QT interval: The interval in milliseconds between the Q wave and T wave on an electrocardiogram
